# Scientists Are Engineering Asphalt That Is Safer for Humans and the Environment

**DOI:** 10.1021/acscentsci.3c00653

**Published:** 2023-06-07

**Authors:** Payal Dhar

Asphalt is used for millions of kilometers of roads globally, as well as for sidewalks, roofs, parking lots, and other outdoor areas. It is used for waterproofing and soundproofing and in construction and manufacturing. On top of that, it’s cheap, easy to repair, and 100% recyclable.

But if you’ve ever smelled fresh asphalt on a newly laid road and imagined your life being shortened by a couple of days, that may not be too far from the truth. Recent explorations into the volatile emissions from asphalt are beginning to show that roads release chemicals that can be harmful not just to the environment but also to human health directly.

**Figure:**
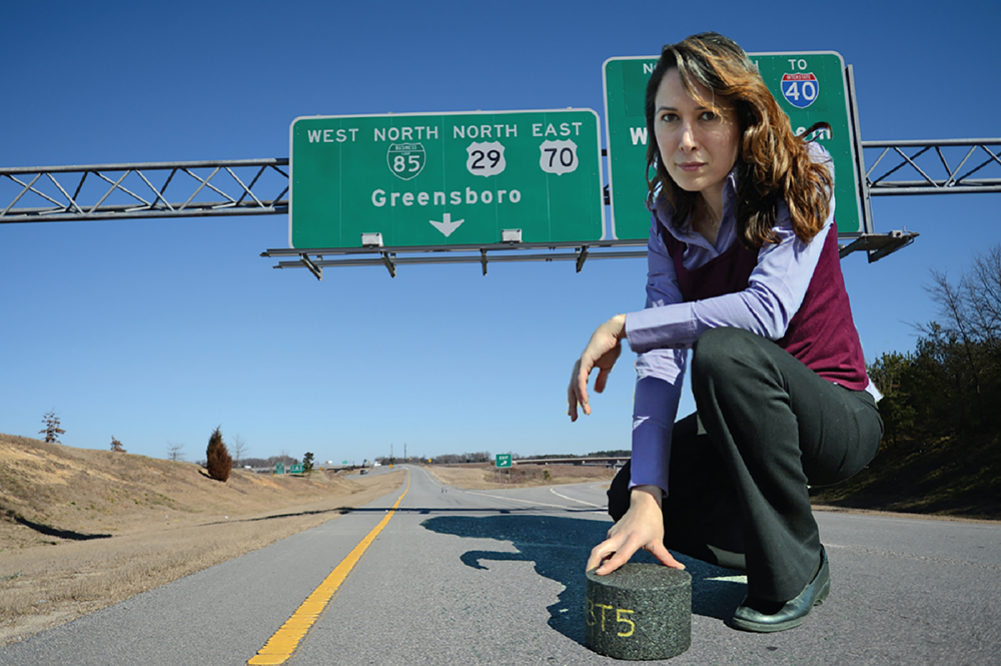
Elham Fini holds a sample of her lab’s biochar-enhanced asphalt. Credit: Charles Watson.

Asphalt is not a single substance. It is a mixture of many chemicals, and there are thousands of different asphalt mixes designed for different budgets, levels of traffic, environments, climates, and existing surface structures. As a fossil fuel product whose manufacture requires temperatures of up to 350 °C, the material has a not-insignificant carbon footprint. According to the trade group National Asphalt Pavement Association, between 2009 and 2019, U.S. greenhouse gas emissions from the manufacture of asphalt mix hovered at around 20 million metric tons of carbon dioxide equivalent annually—which in 2019 would have amounted to about 0.3% of the U.S. total of such emissions. (For comparison, the U.S. Environmental Protection Agency estimated that commercial air transportation contributed about 2.1% of U.S. emissions in the same year.) This estimate does not account for other stages of asphalt’s lifecycle such as transport, installation, and disposal.

Asphalt’s impact on human health is a relatively new topic of study. Recent research suggests that asphalt surfaces can emit particulate air pollution and other volatile compounds that are hazardous to humans. Because asphalt consists of a complex combination of tens of thousands of chemicals, correlations between exposure and health effects are difficult to measure.

Strategies to minimize the negative impacts of roads and other asphalt surfaces have traditionally focused only on the environmental aspect. But having defined links to human health, some researchers now believe that making the next generation of asphalt will be a balancing act between mitigating carbon footprint, enhancing durability, and limiting adverse health outcomes for people paving and living near the roads. They are exploring asphalt additives such as nonfossil-derived components and devising mixes that better withstand what traffic and nature throw at them—all to keep volatile pollutants from entering the air we breathe.

Traditionally, asphalt binder is made from sticky, viscous asphaltenes—bottom-of-the-barrel residues left when gasoline, diesel, jet fuel, and other compounds are removed from crude oil during the refining process—and lighter maltenes. While we usually refer to paving material as “asphalt,” only about 5% of the jet-black mixture dumped on roads is true asphalt. It binds the other 95%, which is made up of gravel and other aggregates.

After this chemical blend is laid on roads, it is barraged by heat, sun, and other weather conditions, as well as the weight of traffic, until it breaks down into smaller, lighter molecules. The heat can coax these molecules to vaporize and float off, producing that pungent smell we all know.

The emissions comprise potentially hazardous volatile organic compounds (VOCs) such as oxygen- or sulfur-carrying aromatics, including benzofuran, benzoic acid, dibenzothiophene, hexanethiol, and polycyclic aromatic hydrocarbons (PAHs). Some VOCs can irritate the eyes, nose, and throat, damage nerves and other organs, and possibly cause cancer, according to the American Lung Association. PAHs have also been linked to blood and liver problems.

“There are emissions from asphalt products over a range of temperatures,” even “pretty modest” ones, says Albert Presto, a chemical engineer at Carnegie Mellon University. His team, along with Drew Gentner’s group at Yale University, evaluated asphalt emissions as an unaccounted-for source of pollution in urban air quality calculations. The researchers heated fresh asphalt to different temperatures, exposed it to simulated sunlight, and found that the material was emitting a mélange of PAHs, alkanes, and aromatic compounds. They saw a 300% jump in VOC emissions when they exposed the asphalt to moderate solar radiation and a 70% increase in those emissions for every 20 °C that they cranked up the ambient temperature.

Occupational exposure is the most obvious threat as VOCs waft out of newly laid pavement and crews are regularly subjected to high-concentration emissions. But “more broadly, across a whole city, you have lots of [asphalt] surfaces emitting at a low level for a long time,” Presto says. “That’s ultimately contributing to secondary aerosols”—organic chemicals that undergo further oxidation once they’re airborne and agglomerate into particulate matter.

Even if these surfaces’ contributions to the total aerosol load is small, they should be monitored, because they continue for a long time and can potentially affect a lot of people. Measuring human exposure to asphalt is only half the problem. Researchers and regulators also don’t know all the potential health impacts of the material’s myriad components, individually or in combination, says Arizona State University’s Judith Klein-Seetharaman, who studies computational protein biochemistry.

As things stand, determining unhealthy exposure to many of the compounds found in asphalt depends on a list of reference compounds and their accepted exposure limits. But those limits were calculated according to what could be practically detected using spectrographic and chromatographic analysis and surface-sampling methods at the time, which in some cases was in the early 1990s. These approaches don’t provide enough information to draw conclusions about how these chemicals might react in combination, according to Klein-Seetharaman. “It’s an oversimplification,” she says, because analysis has shown that asphalt emissions comprise several thousand compounds, including some whose health outcomes haven’t even been measured.

Klein-Seetharaman would also like studies to account for the long-term accumulation of these chemicals in the human body. She notes the possibility of secondary systemic effects, in which VOCs can initially enter a person’s system via inhalation, for example, and be transported through the bloodstream to other organs. Some of these pollutants can linger, hidden inside lipid droplets, and emerge into the bloodstream when the lipids are metabolized years later, causing long-term effects.

To get a better sense of the web of interactions, Klein-Seetharaman and her colleagues reviewed literature documenting the effects of known compounds in asphalt and their cellular biomarkers. They mapped interconnections between these pollutants, the various genes affected by those pollutants, and the potential health effects, including cardiovascular disease, liver damage, asthma, chronic obstructive pulmonary disease, and skin conditions.

Many researchers believe that most asphalt emissions occur during the construction phase, but Elham Fini, one of Klein-Seetharaman’s collaborators at Arizona State, points out that she smells asphalt fumes in the desert summer year after year. This indicates that paved surfaces are continuing to degrade.

Fini’s lab is working toward a solution by making an asphalt binder that emits fewer harmful chemicals. In doing so, her team also wants to find ways to keep asphalt’s carbon footprint in check. The researchers are investigating biomass-derived additives as a low-carbon option. These are inherently carbon sinks and can grab VOCs before they float off into the air. One of these materials is iron-rich biochar, which comes from the thermochemical conversion of waste biomass like algae and manure. Biochar is a carbonaceous material that has been used for CO_2_ capture and environmental cleanup because its highly porous structure can trap gas and heavy-metal molecules. Fini and her colleagues found that introducing an iron-rich version of biochar to asphalt resulted in a 76% reduction in VOC emissions, versus 59% with regular biochar. In computer simulations, the team saw that functional groups containing iron–nitrogen bonds could efficiently adsorb and catalytically degrade VOCs.

The team is also working on a material derived from liquefied algae biomass and adding it to asphalt to snatch organic compounds that are precursors to VOCs and secondary aerosol pollutants before they become airborne. The researchers have dubbed the biomass production process AirDuo because they do it in two steps: they employ carbon capture technology to harvest CO_2_ from air and then feed that CO_2_ to algae or some other biological material. When mixed into asphalt, the resulting binder selectively adsorbs and retains various reactive pollutants and their precursors. In other words, the asphalt cleans up after itself.

AirDuo can be tailored to remove volatiles not just from asphalt but from other sources, such as refineries or car exhausts. “Wherever you get to sequester carbon and prevent it from going back to the air, it’s good,” Fini says. Her team is working on scaling up the technology.

Lab experiments show that, apart from controlling air quality, Fini’s additives increase the durability of roads. This is yet another force to balance when designing new asphalt mixes: if roads are more resilient, their breakdown can be slowed, and they do not have to get repaved as often. That leads to lower asphalt consumption and the potential reduction of VOC and PAH releases.

Andrew Barron, a chemical engineer at Swansea University, is applying nanotechnology to this fundamental issue. As it weathers, asphalt breaks down chemically, but it also breaks down physically. That fragmentation leads to more exposed surfaces, which causes additional degradation into VOCs. Water exacerbates this process as it flows into cracks and further breaks up the asphalt.

A study from Barron’s lab reported that adding engineered clay or silica nanoparticles to asphalt binders helps alleviate degradation and extends a road’s lifetime. The nanoparticles coat the asphalt, creating a composite material that acts as a shield against oxidation, heat, and water. The heat resistivity prevents the decomposition of chemical bonds in the asphalt, Barron says, thus reducing cracking and the consequent emissions normally coming off roads. Under high-temperature, -pressure, and -ultraviolet-light conditions, asphalt mixes that contained 0.2–0.3% of the nanoparticles by weight retained 1.5–2 times as much viscosity as normal asphalt, a proxy for aging.

Barron is optimistic about commercializing the technology. The materials his team needs to make its nanoparticles are already being manufactured at a large scale, he says. And because the nanoparticles can be mixed into asphalt on-site, the additive would not require any changes in the road-laying process.

Shenghua Wu, a civil engineer at the University of South Alabama, notes that what works great in the lab may not be practical on the ground. “We can have a design idea, but the people who are going to make that happen have to feel comfortable about making the change.”

Richard Willis, vice president of engineering, research, and technology at the National Asphalt Pavement Association, admits that the industry is not necessarily the quickest to adapt to new technologies, mostly because of issues related to economies of scale—it takes a long time to swap out every kilometer of asphalt.

Wu says that collaboration between labs, companies, and government agencies is key. Achieving carbon-neutral goals will require a lot of work, he says. “Even though I notice a lot of resistance and delays, as long as you continue sharing knowledge, I have hope that we can push our industry...in a more sustainable direction.”

Fini adds that sustainability is about more than carbon emissions, though, and factoring in health is essential: “I can use something that has a lower carbon footprint, but it could degrade and release toxic compounds to the air that we breathe.” Breathing in CO_2_ from asphalt might give you a headache, but breathing in the same amount of benzothiophene could be much, much worse, she says.

*Payal Dhar is a freelance contributor to*Chemical & Engineering News, *the independent news outlet of the American Chemical Society*.

